# Deep Learning for Novel Antimicrobial Peptide Design

**DOI:** 10.3390/biom11030471

**Published:** 2021-03-22

**Authors:** Christina Wang, Sam Garlick, Mire Zloh

**Affiliations:** 1 UCL School of Pharmacy, University College London, London WC1N 1AX, UK; christina.wang.19@ucl.ac.uk; 2 Department of Computer Science, The University of Manchester, Manchester M13 9PL, UK; samgarlick@hotmail.co.uk; 3 Faculty of Pharmacy, University Business Academy in Novi Sad, 21000 Novi Sad, Serbia

**Keywords:** antimicrobial peptides, deep learning, long short-term memory, machine learning, peptide design, *Escherichia coli*

## Abstract

Antimicrobial resistance is an increasing issue in healthcare as the overuse of antibacterial agents rises during the COVID-19 pandemic. The need for new antibiotics is high, while the arsenal of available agents is decreasing, especially for the treatment of infections by Gram-negative bacteria like *Escherichia coli*. Antimicrobial peptides (AMPs) are offering a promising route for novel antibiotic development and deep learning techniques can be utilised for successful AMP design. In this study, a long short-term memory (LSTM) generative model and a bidirectional LSTM classification model were constructed to design short novel AMP sequences with potential antibacterial activity against *E. coli*. Two versions of the generative model and six versions of the classification model were trained and optimised using Bayesian hyperparameter optimisation. These models were used to generate sets of short novel sequences that were classified as antimicrobial or non-antimicrobial. The validation accuracies of the classification models were 81.6–88.9% and the novel AMPs were classified as antimicrobial with accuracies of 70.6–91.7%. Predicted three-dimensional conformations of selected short AMPs exhibited the alpha-helical structure with amphipathic surfaces. This demonstrates that LSTMs are effective tools for generating novel AMPs against targeted bacteria and could be utilised in the search for new antibiotics leads.

## 1. Introduction

In the past few decades, antimicrobial resistance has become an increasingly urgent challenge in healthcare [1], with antimicrobial-resistant infections estimated to increase to 10 million cases annually by 2050 [2]. In light of the 2020 COVID-19 pandemic [3], additional concerns have been expressed, due to the increase in antimicrobial drug prescriptions [4,5] that may unintentionally potentiate the development and the spread of antimicrobial resistance [6]. Additionally, the current pipeline for novel antibiotics is unable to meet the challenges that multidrug-resistant superbugs pose [7].

A highly promising approach to this problem is in the development of drugs based on antimicrobial peptides (AMPs). AMPs are naturally present in the innate immune system and have broad-spectrum antimicrobial properties aiding in the defence against invading microorganisms [8,9]. They are usually short cationic peptides of up to 100 amino acids [9], that often adopt an alpha-helical secondary structure with amphiphilic surface properties, regarded as essential for establishing antimicrobial activity [10,11,12]. AMPs’ main mechanism of action is the disruption of the target microorganism’s cell membrane, through hydrophobic or electrostatic interactions, causing lysis of the cell [13]. AMPs offer several advantages over conventional small molecule antibiotics, including rapid killing of bacteria with broad-spectrum activity, antimicrobial immunomodulatory effects, and a lower likelihood for antimicrobial resistance to develop [12,14,15].

Currently, various databases have been developed that contain information about AMPs and their activities, including A Database of Anti-Microbial Peptides (ADAM) [16], Antimicrobial Peptide Database (APD) [17], Collection of Anti-Microbial Peptides (CAMP) [18], Database of Antimicrobial Activity and Structure of Peptides (DBAASP) [19], Data Repository of Antimicrobial Peptides (DRAMP) [20], Giant Repository of AMP Activities (GRAMPA) [21], a database for Linking AMPs (LAMP) [22], and Yet Another Database of Antimicrobial Peptides (YADAMP) [23]. Considering the vast amount of AMP sequences available, machine learning is a method that is often used to identify promising AMPs. While shallow machine learning methods, such as support vector machine, k-nearest neighbour, random forest, and multilayer perceptron, are utilised in the classification of AMP sequences successfully [24,25,26,27,28,29,30,31,32,33,34,35,36], there is an increasing number of studies that are employing a deep learning approach [21,37,38,39,40,41]. The main advantage of deep learning, especially in the era of big data [42], is the capability to automatically extract commonalities and complex features from large amounts of raw data. Deep learning also reduces the need for feature engineering, which requires expert knowledge in the subject’s domain [42]. Long short-term memory (LSTM) models are a popular type of recurrent neural network and have led to successes in several studies on AMP classification [37,40,41,43] and generation [41,43]. A variation of the LSTM, the bidirectional LSTM, is often used in natural-language processing [44,45] and other order-dependent problems, including the classification of AMP sequences [43].

Despite these successful applications, to the best of our knowledge, none have focused on designing AMPs with activity against specific bacteria species. The majority of novel antibacterial agents in development target Gram-positive bacteria, while Gram-negative bacteria are in critical need of new treatments [7,46]. Therefore, in this study, we focus on generating AMPs that target *E. coli*, a Gram-negative ESKAPE bacterium [47] that is considered a high priority antibiotic resistance threat [48,49]. Additionally, the design step was focused on generating short AMPs with a maximum sequence length of up to 20 residues, while maximising the potential antimicrobial activity of the designed AMPs by training the models using sequences with proven low minimal inhibitory concentrations (MICs). Such peptides are easier and less expensive to manufacture [12,14]. To achieve the above objectives, two negative data sets were established, including one comprising sequences from AMP databases found to be inactive against *E. coli*. Additionally, Bayesian hyperparameter optimisation was implemented and the decision boundary was moved to an optimum using receiver operating characteristic (ROC) curves.

**Figure 1:**
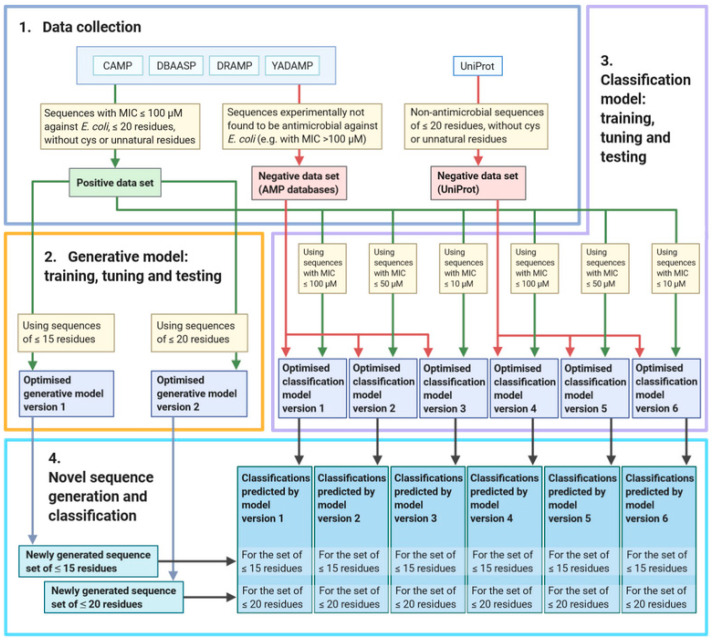
Workflow for the machine learning process. (**1**) Data for the positive set were collected from antimicrobial peptide (AMP) databases Collection of Anti-Microbial Peptides (CAMP), Database of Antimicrobial Activity and Structure of Peptides (DBAASP), Data Repository of Antimicrobial Peptides (DRAMP), and Yet Another Database of Antimicrobial Peptides (YADAMP). Two negative data sets were collected, from AMP databases and UniProt, respectively. (**2**) Two versions of the generative model were trained, tuned, and tested on the positive data set. (**3**) Six versions of the classification model were trained, tuned, and tested on the positive data set, as well as either the UniProt or AMP negative data set, using different minimal inhibitory concentration (MIC) cut-offs. (**4**) The optimised versions of the generative model ultimately produced two sets of AMP sequences. These sequences were then classified by the six optimised versions of the classification model, resulting in 12 sets of predictions.

Hence, the aim of this study is to develop a method employing deep learning approaches to design and subsequently classify short de novo antimicrobial peptide sequences with potentially high antibacterial activity against *E. coli* (Figure 1).

## 2. Materials and Methods

### 2.1. Data Set Collection

Data were collected from four AMP databases: CAMP [18], DBAASP [19], DRAMP [20], and YADAMP [23] (Figure 1). Sequences of linear peptides with antimicrobial activity against *E. coli*, with an MIC of ≤100 µM, and a maximum length of 20 residues, were included in the positive data set. Duplicate sequences from multiple databases were only included once, and the geometric mean of their MIC values was taken if the databases’ records indicated different MICs for the same sequence. Sequences containing cysteine residues or residues other than the 20 naturally occurring amino acids were excluded, considering that potential future synthesis of peptides containing such residues might be difficult [41]. The collected MICs were converted into µM, using an estimate of the molecular weight of the sequence, by taking the sequence’s length and multiplying it by 110 Da, the average molecular weight of an amino acid [50,51].

Two sets of negative controls were generated from sequences collected from UniProt [52] and the previously listed AMP databases. The first set of negative controls was formed by following previously reported approaches [21,37]. A search was performed on UniProt using the search term: “length:[* TO 20] NOT antimicrobial NOT antibiotic NOT antiviral NOT antifungal NOT fungicide NOT secreted NOT secretory NOT excreted NOT effector NOT defensin”. The term length:[* TO 20] filters results to include only sequences with 20 or fewer residues in length. Duplicates and sequences containing cysteines or unnatural amino acids (B, J, O, U, X, and Z) were removed. From the remaining sequences, a number of sequences equal to the number of sequences in the positive data set was chosen at random with the sequence length distribution similar to that of the positive data set. The other negative control set was established from the sequences from the AMP databases with known MIC values against *E. coli* listed as >100 µM or greater. Such sequences could be considered potentially very weakly antimicrobial (the sequence could have yielded some activity if concentrations greater than 100 µM were tested) or non-antimicrobial.

### 2.2. Data Processing

**Figure 2:**
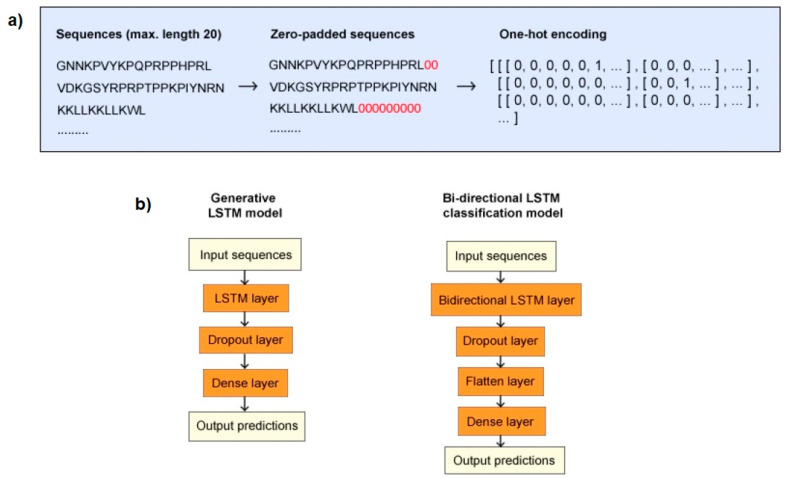
Data processing (**a**) and model structures (**b**). (**a**) Sequences had a maximum length of 20 residues and were zero-padded if their length was under 20 residues. Subsequently, the sequences were one-hot encoded, after which they were ready to be input into the deep learning models. (**b**) The generative long short-term memory (LSTM) model started with an LSTM input layer. A dropout layer was added to reduce overfitting and a dense layer was the last layer, responsible for outputting probabilities. The bidirectional LSTM classification model started with a bidirectional LSTM input layer, after which a dropout layer was applied. A flatten layer was added to reduce the dimensions of the input data, and a dense layer was responsible for the output of predictions.

Sequences were input into the models as a string of letters, with each letter representing a one-letter abbreviation for an amino acid. For the classification model, the available data were split into a training/validation set and a test set, which evaluates how well the model would perform on new, unseen data [53]. The training data for the classification model were oversampled after data partitioning, to account for the data imbalance. In order to homogenise the input data, all the sequences used for training and testing were padded with zeros up to the maximum sequence length, as LSTM models take inputs of the same length and dimension (Figure 2) [54]. The peptide sequences were then shuffled using a seed, one-hot encoded, and represented as 3D tensors (with three axes for the number of peptide sequences, the length of each sequence, and the number of different possible residues).

### 2.3. Model Structures and Training

In order to generate novel AMP sequences and classify them as antibacterial or non-antibacterial, two types of deep learning models, both sequential character-level natural-language processing models, were constructed. The code for the models can be found in the Supplementary Materials.

#### 2.3.1. Generative Model

For the purpose of generating new sequences and the design of novel AMPs, a generative model comprising three layers was constructed (Figure 2). The LSTM layer was utilised as the input layer to extract features across time steps, which in this study corresponded with each residue in each sequence. A dropout layer was added to prevent the model from overfitting. The output layer was a dense layer with a softmax activation function, enabling the model to output the probabilities for each residue character to be the next character in the sequence. For the compilation of the model, categorical_crossentropy was used as the loss function and RMSprop was used as the optimiser. The Keras early stopping callback function was implemented to stop the training once the model stopped improving. Two versions of the generative model were trained using the positive data set, one using sequences with a length of 15 residues and under, the other on 20 residues and under. The training data were defined as each residue excluding the last residue of the sequence, while the targets were the respective next residues for each position. For example, for the sequence VDKGSYRPRPTPPKPIYNRN, VDKGSYRPRPTPPKPIYNR was the training data, and DKGSYRPRPTPPKPIYNRN the target.

#### 2.3.2. Classification Model

To theoretically confirm whether the generated sequences have a potential to act as antimicrobials, a bidirectional LSTM model was built to classify the new peptides. The sequential classification model consisted of four layers (Figure 2). A bidirectional LSTM layer was used as the input layer, in order to extract features across time steps in both chronological and antichronological directions of the sequence. To reduce overfitting, a dropout layer was applied. A flatten layer was incorporated to reduce the dimensions of the input data. The output layer was a dense layer with a sigmoid activation function, which outputs a binary classification in the range of [0,1]. For the model compilation, binary_crossentropy was utilised as the loss function and RMSprop was used as the optimiser. The Keras early stopping callback function was implemented to stop the training once the model stopped improving. For the classification model, an MIC cut-off was implemented as a parameter, and three cut-offs, ≤100 µM, ≤50 µM, and ≤10 µM, were used to train the model on the positive data with different levels of antimicrobial activity. Two negative control sets were used in conjunction with each of these three cut-offs, resulting in a total of six different versions of the classification model. To train these models, 80% of the input data were labelled as the training set, and the remaining 20% were considered the test set.

### 2.4. Model Tuning

In order to optimise the models’ hyperparameters, Bayesian hyperparameter tuning was applied, in which different learning rates [0.1, 0.01, 0.02, 0.03, 0.001, 0.0001], units per layer [32–512; step 32], and dropout rates [0.0–0.8, step 0.1] were explored. The max_trials was set to 60. For the generative model, the hyperparameter combination with the lowest loss was chosen. For the classification model, Bayesian hyperparameter tuning with 5-fold cross validation was performed. During each fold, a different fifth of the training data were held out for validation, and the average loss and accuracy of the five folds were calculated. The model with the best average accuracy was selected. This optimised model was trained on the entire training set, and subsequently tested on the test set.

### 2.5. Model Evaluation

To evaluate the performance of the six optimised versions of the classification model, ROC curves were plotted and the areas under the curve (AUCs) calculated. The ROC curve plots the true positive rate (TPR) against the false positive rate (FPR) for each decision boundary. The optimal decision boundary was determined from each ROC curve by calculating the Youden index (J):

J = sensitivity + specificity − 1.(1)

Confusion matrices implementing these boundaries were generated, displaying the true positive (TP), true negative (TN), false positive (FN), and false negative (FN) predictions. The sensitivity, specificity, precision, and accuracy of each version of the model were calculated as model performance measures, using the following formulas:

Sensitivity = TPR = TP/(TP + FN),(2)

Specificity = TN/(TN + FP),(3)

Precision = TP/(TP + FP),(4)

Accuracy = (TP + TN)/(TP + FN + TN + FP)(5)

### 2.6. Data Generation and Classification

Two optimised versions of the generative model were used to sample two sets of 5000 new sequences with, respectively, maximum sequence lengths of 15 and 20 residues. A temperature factor of 1.0 was introduced during sequence sampling. For each sampled sequence, the first residue was randomly selected. Each following residue was determined by incorporating the probabilities that were predicted by the generative model, and the randomness introduced by the temperature, which prevented overly repetitive sequences from being generated. This sampling process was continued for each sequence up to the maximum sequence length. In this way, two sets of 5000 generated sequences were obtained, with a sequence maximum length of either 15 or 20 residues. Duplicates and sequences that were identical to the input training sequences were removed from each set.

The six optimised versions of the classification model were then used to classify the two sets generated with the generative model. Instead of utilising the default decision boundary of 0.5 (an output greater than 0.5 classifies the sequence as antimicrobial, otherwise it is classified as non-antimicrobial), the threshold is moved to the optimal decision boundary for each version of the classification model. The percentages of generated sequences that were classified as antimicrobial were then calculated.

### 2.7. Further Analysis and 3D Structure Generation

From the generated sequences, a set of selected sequences was taken forward in a case study. Peptides with a sequence length of eight residues were taken from the generated set with a maximum sequence length of 15 residues. Only the peptides that were classified as antimicrobial by all six versions of the classification model were included. Additional predictions for these sequences were generated using an external online tool, AMP scanner (Version 2) [37]. Sequences that were classified as antimicrobial by this tool were taken forward. The sequences were then uploaded to ToxinPred [55], an online tool used to predict the toxicity of peptides. The non-toxic peptides were taken forward for 3D structure generation. PEP-fold 3 [56] was used to predict the 3D structures. The 3D structures were then loaded into Chimera (Version 1.14) [57], which was used for visualisation purposes and for the generation of hydrophobicity surfaces. Finally, a search was performed in various databases (ADAM [16], APD [17], CAMP [18], DBAASP [19], DRAMP [20], YADAMP [23], UniProt [52]), to confirm whether these peptide sequences are novel.

### 2.8. Experimental Setup

The models were built in Python using Keras (Version 2.3.1, running on GPU) [58] with a TensorFlow back end (Version 2.1.0) [59]. The models were trained on a computer with an NVIDIA GeForce GTX 970 GPU. The Keras Tuner was implemented to optimise the hyperparameters using Bayesian hyperoptimisation [60]. The MATPLOTLIB library was imported for data visualisation [61]. The scikit-learn [62] and pandas [63] libraries were also utilised.

## 3. Results

### 3.1. Data Sets

At the moment of data collection in November 2020, the CAMP, DBAASP, DRAMP and YADAMP databases, respectively, contained 8164, 15,809, 20,744, and 2525 AMP sequences. After selecting the sequences with activity against *E. coli* (with an MIC of ≤100 µM) and a length of 20 sequences or under, and excluding duplicates and sequences containing cysteine residues or unnatural residues, the search resulted in a total of 1119 sequences. The positive data set with sequences of length ≤ 15 residues contained 690 sequences. Two sets of negative controls were collected: one containing 1119 sequences from UniProt, selected randomly from the 82,459 sequences found with the search term, and the other containing 142 sequences from the AMP databases and literature [64]. The sequences had lengths ranging from two to 20, with an average of 14 residues (Figure S1). The two versions of the generative model were trained on the positive data sets containing, respectively, 690 and 1119 sequences. For the classification models, the same 1119 sequences from the positive data set were utilised, as well as the negative control sequences from either the UniProt set (1119, 1040, or 692 sequences, for MIC cut-offs of 100, 50, and 10 µM, respectively) or the AMP database set (142 sequences, oversampled). The data sets are available in the Supplementary Materials.

### 3.2. Classification Model Tuning, Evaluation and Validation

**Figure 3:**
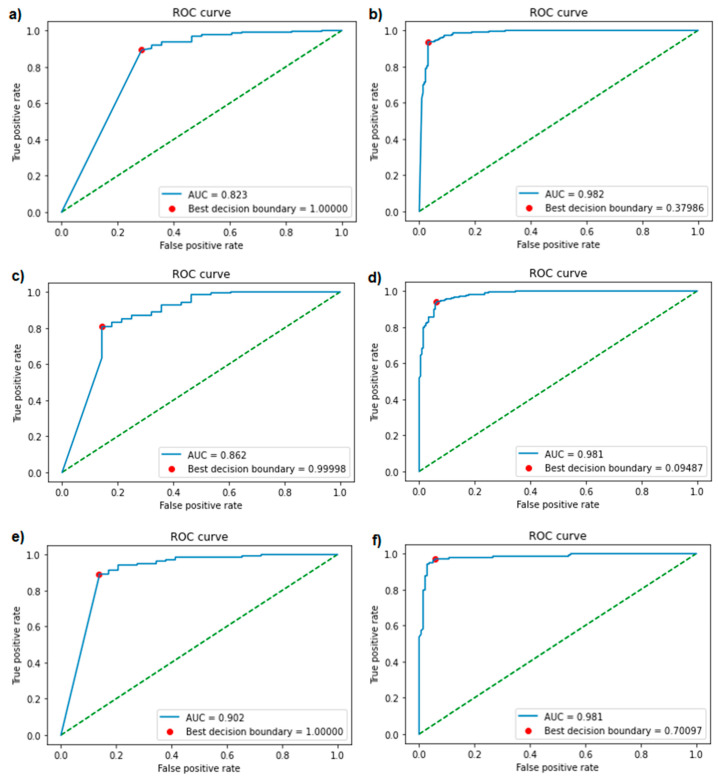
Receiver operating characteristic (ROC) curves and areas under the curve (AUCs) of each version of the classification model. The best threshold is represented by a red dot. The left three figures (**a**,**c**,**e**) are from the models trained with negatives from antimicrobial peptide (AMP) databases (model Version 1–3), the right three (**b**,**d**,**f**) with negatives from UniProt (model Version 4–6). From top to bottom, the models were trained with a minimal inhibitory concentration (MIC) cut-off of 100 (**a**,**b**), 50 (**c**,**d**), and 10 µM (**e**,**f**). The green diagonal line represents the no-discrimination line, where the model is of no use, and the false positive rate is equal to the true positive rate.

**Figure 4:**
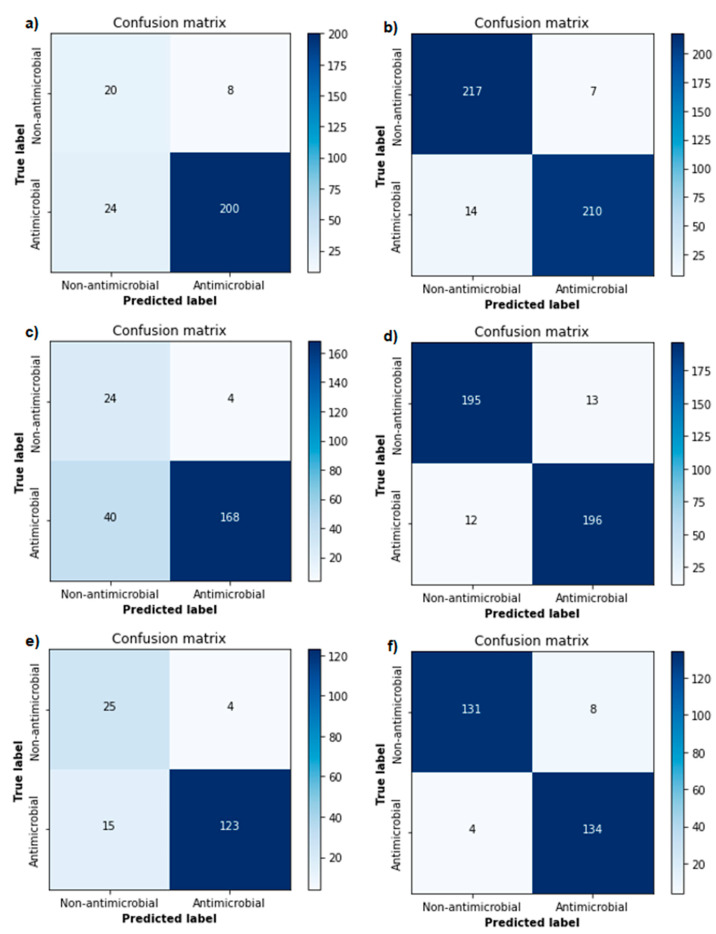
Confusion matrices of each version of the classification model. The left three figures (**a**,**c**,**e**) are from the models trained with negatives from antimicrobial peptide (AMP) databases (model Version 1–3), the right three (**b**,**d**,**f**) with negatives from UniProt (model Version 4–6). From top to bottom, the models were trained with a minimal inhibitory concentration (MIC) cut-off of 100 (**a**,**b**), 50 (**c**,**d**), and 10 µM (**e**,**f**).

Using Bayesian hyperparameter optimisation, the best parameter combinations were determined for each version of the generative and classification models. An overview of the optimised parameters can be found in the supplements (Tables S1 and S2). The validation accuracies of the different versions of the classification model ranged from 81.6% to 88.9% (Table 1). ROC curves were plotted and AUCs (ranging from 0.823 to 0.982) were calculated for each version of the classification model (Figure 3), showing excellent performance in each of the models. Optimal decision boundaries were calculated and implemented in the generation of the confusion matrices (Figure 4). The calculated performance measures for each version of the classification model are displayed in Table 1. The sensitivity (80.8–97.1%), specificity (71.4–96.9%), precision (93.8–97.7%), and accuracy (81.4–95.7%) showed a strong performance from each version of the classification model, indicating a good recognition of AMPs. 

### 3.3. Novel Sequence Generation and Classification

After filtering out duplicates and the sequences that only contained a single residue, the set with a sequence length of ≤15 residues contained 678 sequences. The set with a sequence length of ≤20 residues contained 1832 sequences. The predicted classifications for the two novel sequences sets, by each version of the classification model, are displayed as percentages of antimicrobial peptides in Table 2.

### 3.4. Further Analysis and Generated 3D Structures

**Figure 5:**
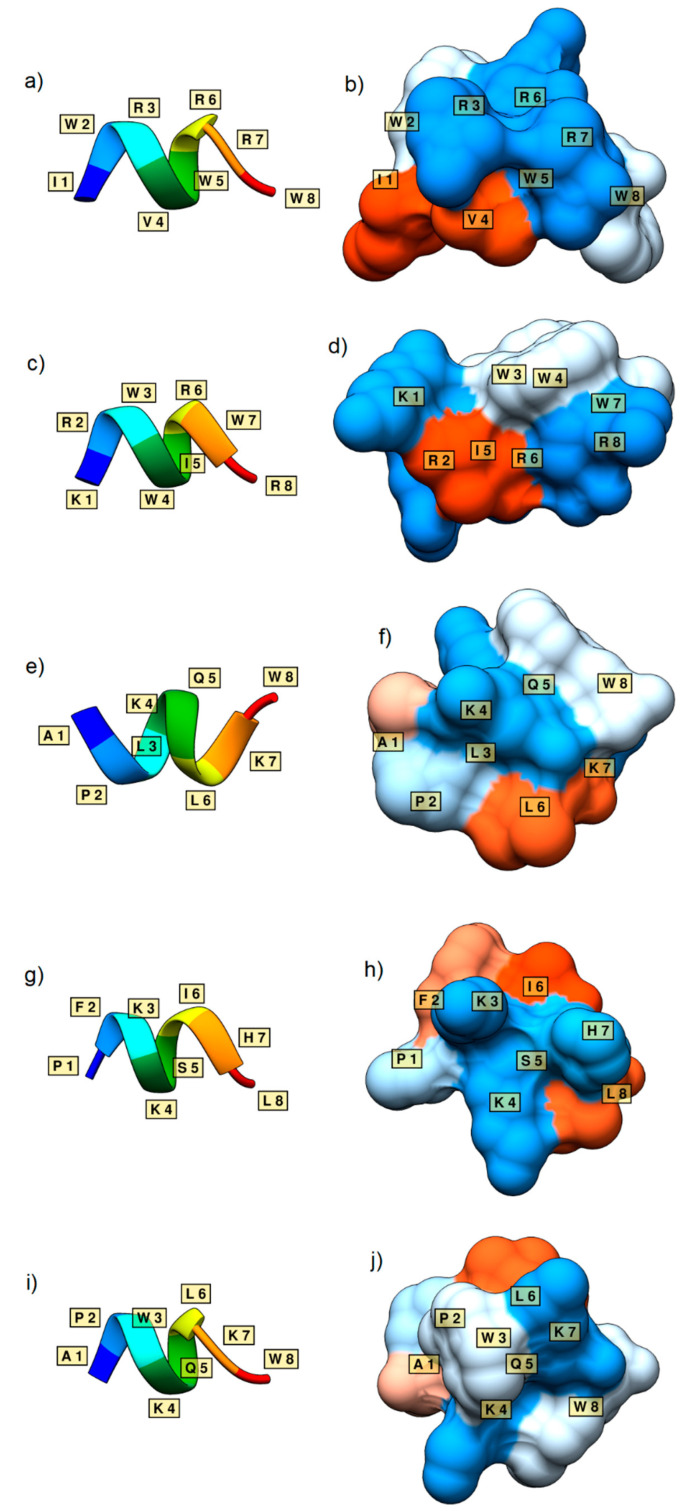
Secondary structures and surface representations of sequences IWRVWRRW (**a**,**b**), KRWWIRWR (**c**,**d**), APLKQLKW (**e**,**f**), PFKKSIHL (**g**,**h**), and APWKQLKW (**i**,**j**). The residues are labelled according to their one-letter abbreviation. Surface representations show the hydrophilicity (blue) and hydrophobicity (orange) of the peptides. White surfaces represent a hydrophobicity of around 0.0.

As a case study, 14 novel sequences with the length of eight residues, classified as antimicrobial by all six versions of the classification model, were taken from the generated AMP sets (Table 3). Twelve of these sequences were labelled as antimicrobial by the external predictor AMP scanner. All 12 were considered non-toxic by ToxinPred, and therefore were taken forward into 3D structure prediction. Five of the sequences containing alpha helical structures, *IWRVWRRW*, *KRWWIRWR*, *APLKQLKW*, *PFKKSIHL*, and *APWKQLKW*, are shown in Figure 5. 3D generations generally showed clear alpha-helical secondary structures, and generated surfaces displayed the amphipathic character of each sequence, although the peptide of sequence KRWWIRWR may not be considered truly amphipathic. Furthermore, these exact sequences were not found in various databases (ADAM, APD, CAMP, DBAASP, DRAMP, YADAMP, UniProt), confirming that the newly generated sequences by our models are unique. However, very similar sequences with known antimicrobial activity against *E. coli* were found in the databases, such as the patented sequence IWRVWRRWK from DRAMP [20], and sequences APRKQLKW, APKKQLKW, PFKISIHL, APRKQLKW, and APKKQLKW from YADAMP [23]. This indicates that it is highly likely that our *de novo* sequences will have antimicrobial activity against *E. coli* as well.

## 4. Discussion

As demonstrated in this study, our models based on LSTMs and bidirectional LSTMs can successfully be utilised for the generation and classification of novel AMPs with antibacterial activity against *E. coli*. To the best of our knowledge, our study is one of the first to demonstrate the application of LSTM models on the design of short AMPs targeting a specific bacterial species. The ROC curves, AUCs, and performance measurements displayed our models’ strong ability to discern non-AMPs from AMPs. The peptides that were classified as positive by the models with a ≤10 µM MIC cut-off are especially promising. These were regarded as potent AMPs, considering that common treatments for *E. coli*, such as amoxicillin and gentamicin, have susceptibility breakpoints around similar MIC values [65,66,67,68].

A direct comparison of our models’ performance with those of known studies is difficult, due to the specific targeting of *E. coli* by our models and the more general approach of other published models. However, we can confirm that the performance measures of our classification models (accuracy 81.4%–95.7%, sensitivity 80.8–97.1%, specificity 71.4–96.9%, AUC 82.3–98.2%) are comparable to the performance achieved by previously reported state-of-the-art models (accuracy 83.92–97.0%, sensitivity 82.98–96.2%, specificity 79.92–97.8%, AUC 84.06–97.23%) [18,21,24,26,32,37,39] (Table S3). Furthermore, a selected set of novel sequences was subjected to AMP classification by six other external tools, where excellent agreement with our classification was obtained (85% to 100% of tested sequences were predicted to have potential antimicrobial activity by external tools) (Table S4).

Additionally, 3D structures and surfaces of the newly generated AMPs showed similarities with typical AMP amphipathic short alpha-helix structures [10,11]. However, some of the generated structures lacked an amphipathic character or alpha-helical structure, despite being classified as antimicrobial by the models. It would therefore be beneficial to incorporate these features when developing future models.

Moreover, we proposed an alternative negative data set to the often-used negative controls from UniProt, that are not experimentally confirmed to be non-antimicrobial. As there is currently no database containing experimentally confirmed non-antimicrobial peptides, those sequences would be hard to acquire, especially in the large quantities that deep learning benefits from [53]. We therefore opted to try a smaller second negative data set, containing sequences from AMP databases, that were not found to be antimicrobial in performed experiments. We were, however, not able to collect enough sequences for this negative set to match the size of our positive set. Ideally, a 1:1 split between positive and negative data should be achieved, in order to prevent a bias of the model towards the majority class [69], which can result in the misclassification of the minority data [70]. By oversampling the negative AMP control set, which involves the replication of the negative sequences to make up for the deficit [71], this issue was mitigated. Discovery and collection of more AMP sequences in future research could possibly improve our model, as well as enable AMP design for high-urgency species that we could not collect a sufficient amount of data for, such as carbapenem-resistant *Acinetobacter baumannii* and *Pseudomonas aeruginosa* [47,48,49], or multidrug-resistant *E. coli* [48,49].

A major strength of this study is the choice to generate shorter peptides, with a length of ≤ 20 residues. Shorter peptides are easier and less expensive to manufacture, and thus provide a chance at a wider future clinical application [12,14]. Moreover, while sequences with a length of ≤20 residues will likely form a simple helical pattern, peptides with a length of >30 residues may form complex tertiary structures. A deep learning model that is optimised for sequential residues might not be able to properly account for the complex 3D structure of such a peptide [43].

We also demonstrated an optimised model performance by using Bayesian hyperparameter optimisation and decision boundary shifting. We noticed that few studies in similar past research used automated hyperparameter optimisation for their models, instead opting for a less optimal manual approach. Manual hyperparameter tuning is disadvantageous, because the number of different hyperparameters one can try is more limited, and the effect of tuning specific hyperparameters is hard to predict [72]. Reproducibility is also flawed in manual hyperparameter searches [72,73]. Bayesian hyperparameter tuning is considered more advantageous than grid search or random search, two other popular automated search methods, due to its ability to take the performance of past hyperparameter combinations into account when selecting new hyperparameters to test. This makes it a more efficient method that can yield an even better performance [72,74].

Additional future research could consider the use of alternative deep learning architectures, such as generative adversarial networks (GANs), which have recently been utilised to generate AMPs with high antimicrobial activity by Tucs et al. [75]. Furthermore, as natural-language processing has been predominantly moving towards the use of transformers in recent years [76], transformers such as GPT-3 could be another promising alternative.

Potential weaknesses of the study are the result of the reliance on the information from the AMP databases. The main potential issue is a lack of information on the terminal caps in the peptide sequences. Therefore, all sequences were considered without capping, which leads to designed sequences of uncapped peptides. As the negatively charged C-terminal may adversely affect the interaction of a peptide with the negatively charged membrane of Gram-negative bacteria [11,64], the synthesis of -NH_2_-capped C-terminals should be considered. Additionally, there were some discrepancies in the MIC values reported in the AMP databases for a small set of peptide sequences. However, the use of geometric mean values and consideration of several different MIC cut-off values minimise the potential negative effects of such occurrences.

### Future Perspective in Clinical Application

The obtained results suggest that LSTM deep learning models are promising tools in the search for new antibacterial drug leads and could help to accelerate the process of novel antibiotics discovery. Future synthesis and in vitro testing of the de novo AMPs should be conducted to confirm the in silico results. It should, however, be considered that multiple hurdles must be overcome for an AMP to become an effective antimicrobial drug. The majority of AMPs do not have an optimal activity and need to be improved in terms of pharmacokinetic and pharmacodynamic properties before they can function as therapeutic drugs [9]. AMPs constantly risk degradation during their transport throughout the body, due to, e.g., the intestines, tissue proteases, serum proteases, and clearance by the kidneys [77,78,79], which result in a short serum half-life, limiting their potential for systemic therapeutic use [79]. Another major concern in the clinical use of AMPs is the toxicity, which has not been completely clarified yet [14]. One of the more prominent concerns is toxicity of AMPs against eukaryotic cells, including their haemolytic activity, which can cause lysis in human red blood cells [80]. A practical issue with the clinical application of AMPs is the price. The production of an AMP can range from USD 50 to USD 400 per gram, while traditional antibiotics, such as aminoglycosides, may only cost a fraction of that (USD 0.8 per gram) [14].

Several solutions to counter these difficulties have been proposed, including N-terminal acetylation or C-terminal amidation of the AMPs [79], using unnatural amino acids at labile sites in the AMP [81,82], and the use of nanoparticles to contain AMPs [83]. A very promising approach is the use of peptidomimetics. Peptidomimetics contain pharmacophores that mimic the AMP’s structure, resulting in similar pharmacodynamic effects, while also overcoming the unfavourable pharmacokinetics, reducing toxicity, and potentially improving the potency [84,85,86]. Additionally, the cost of production is lower in peptidomimetics, due to easier synthesis processes, especially in the case of peptidomimetics based on short AMPs [12,14,86].

A rational design of novel short peptides can aid the overcoming of the above obstacles by tuning their properties and minimising toxicity. Therefore, these AMPs have a potential to play an essential role in the future development of treatments against resistant bacteria. Future models can be refined to consider the design of AMPs that synergistically work with other antibiotics, as this enables the use of lower doses of each antibiotic, limiting toxicities [87], as well as in some cases preventing the development of bacterial resistance [88,89]. Such studies need additional data that include already known examples of promising combinations with chloramphenicol [90,91], and amoxicillin–clavulanate and imipenem [92]. Thus, successfully developed AMPs or peptidomimetics based on AMPs are highly likely to be used alongside our current armoury of conventional antibiotics to combat the rise of antimicrobial resistance.

## Figures and Tables

**Table 1:** Performance measurements of each classification model.

Classification Models (Negative Data Set, MIC Cut-Off)	VAL ACC (%, Using 0.5 Threshold)	AUC	Optimal Threshold	SENS (%)	SPEC (%)	PREC (%)	ACC (%)
Model Version 1 (AMP, ≤100 µM)	88.9	0.823	1.000000	89.3	71.4	96.2	87.3
Model Version 2 (AMP, ≤50 µM)	88.5	0.862	0.999985	80.8	85.7	97.7	81.4
Model Version 3 (AMP, ≤10 µM) 3	88.0	0.902	1.000000	89.1	86.2	96.9	88.6
Model Version 4 (UniProt, ≤100 µM)	85.3	0.982	0.379858	93.8	96.9	96.8	95.3
Model Version 5 (UniProt, ≤50 µM)	82.2	0.981	0.094870	94.2	93.8	93.8	94.0
Model Version 6 (UniProt, ≤10 µM)	81.6	0.981	0.700969	97.1	94.2	94.4	95.7

Note: ACC = accuracy, AMP = antimicrobial peptide, AUC = area under the curve, MIC = minimal inhibitory concentration, PREC = precision, SENS = sensitivity, SPEC = specificity, VAL ACC = validation accuracy.

**Table 2:** Predictions of newly generated AMP sets by each classification model.

Classification Models (Negative Data Set, MIC Cut-Off)	Generated Set with Sequences ≤ 15 Residues (% Antimicrobial)	Generated Set with Sequences ≤ 20 Residues (% Antimicrobial)
Model Version 1 (AMP, ≤100 µM)	84.2	79.2
Model Version 2 (AMP, ≤50 µM)	80.7	76.2
Model Version 3 (AMP, ≤10 µM) 3	72.6	72.5
Model Version 4 (UniProt, ≤100 µM)	86.7	70.7
Model Version 5 (UniProt, ≤50 µM)	91.7	73.6
Model Version 6 (UniProt, ≤10 µM)	78.5	70.6

Note: AMP = antimicrobial peptide, MIC = minimal inhibitory concentration.

**Table 3:** Further analysis results of the 14 antimicrobial peptide (AMP) sequences in the case study.

Sequence	Predicted Class by AMP Scanner	Prediction by ToxinPred	Alpha-Helical Structure?	Amphipathic Surfaces?	Novel?
RIHVIRWR	AMP	Non-toxic	No	Yes	Yes
IWRVWRRW	AMP	Non-toxic	Yes	Yes	Yes
APKNQLKW	Non-AMP	-	-	-	-
HRWWRWWR	AMP	Non-toxic	Yes	No	Yes
IRRWRRIW	AMP	Non-toxic	No	No	Yes
PYKISIHL	Non-AMP	-	-	-	-
KRWWIRWR	AMP	Non-toxic	Yes	No	Yes
APRRNVRW	AMP	Non-toxic	No	No	Yes
PFKISIHH	AMP	Non-toxic	No	No	Yes
RRKRWWRR	AMP	Non-toxic	Yes	No	Yes
APLKQLKW	AMP	Non-toxic	Yes	Yes	Yes
PFKKSIHL	AMP	Non-toxic	Yes	Yes	Yes
APWKQLKW	AMP	Non-toxic	Yes	Yes	Yes
RRRRFRRR	AMP	Non-toxic	No	Yes	Yes

## Data Availability

Data used for training and testing the models can be found in the Supplementary Materials.

## Supplementary Materials

The following are available online at https://www.mdpi.com/2218-273X/11/3/471/s1, Code S1: Code for generative model, Code S2: Code for classification model, Figure S1: Histogram of the length distribution of the positive data set, Figure S2: Histogram of the MIC distribution of the positive data set, Figure S3: Histogram of the length distribution of the negative UniProt data set, Figure S4: Histogram of the length distribution of the negative AMP data set, Table S1: Generative model hyperparameters optimised with Bayesian hyperparameter optimisation, Table S2: Classification model hyperparameters optimised with Bayesian hyperparameter optimisation, Table S3: Performance comparison of our classification models with other state-of-the-art machine learning models, Table S4. Predictions of the 14 AMP (antimicrobial peptide) sequences in the case study, by external AMP classification tools.
