# Supplementary material for: Deep Learning for Novel Antimicrobial Peptide Design
Source: Biomolecules. 2021 Mar 22;11(3):471. doi: 10.3390/biom11030471 (PMC8004669; doi:10.3390/biom11030471)
Supplement: Supplementary file 1 [file biomolecules-11-00471-s001.pdf]

Supplementary Material

Deep Learning for Novel Antimicrobial Peptide Design

Christina Wang [1], Sam Garlick [2] and Mire Zloh [1, 3,*]

[1] University College London, UCL School of Pharmacy, London WC1N 1AX, UK
[2] Department of Computer Science, The University of Manchester, Manchester, M13 9PL, UK
[3] Faculty of Pharmacy, University Business Academy in Novi Sad, Novi Sad, Serbia
[*] Correspondence: m.zloh@ucl.ac.uk

Contents

Codes 2
Figures 3
Tables 5
References 8

Codes

Codes for both generative and classification models are available from authors by request to Christina Wang (christina.wang.19@ucl.ac.uk).

Figures

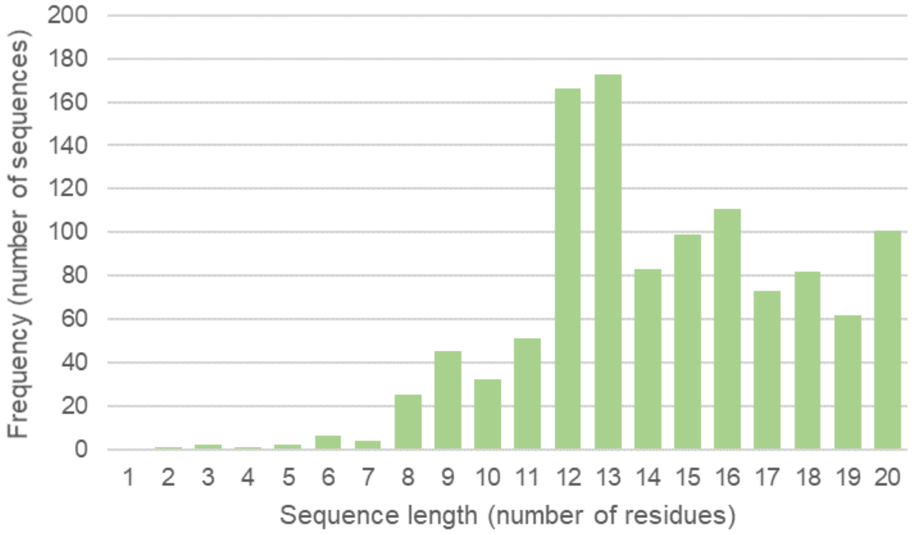

Figure S1. Histogram of the length distribution of the positive data set (n = 1119).

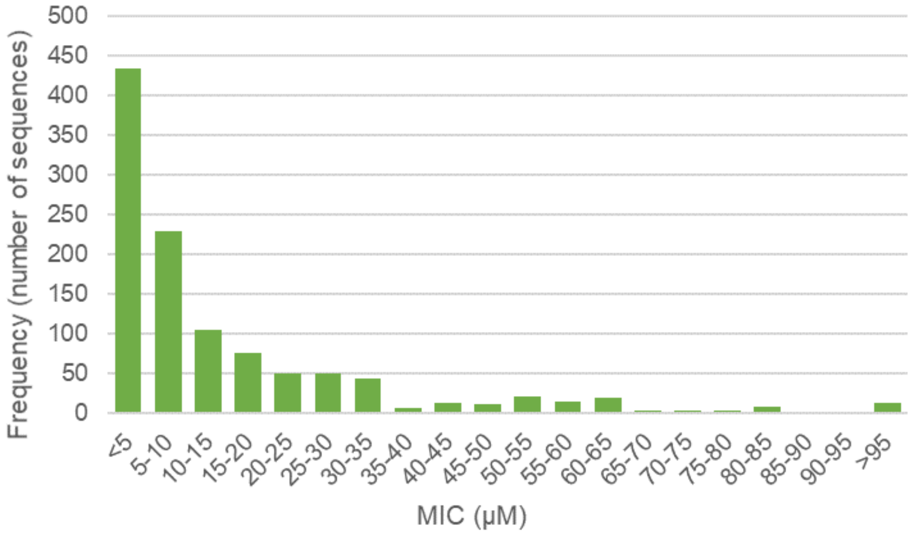

Figure S2. Histogram of the MIC (minimal inhibitory concentration) distribution of the positive data set (n = 1119).

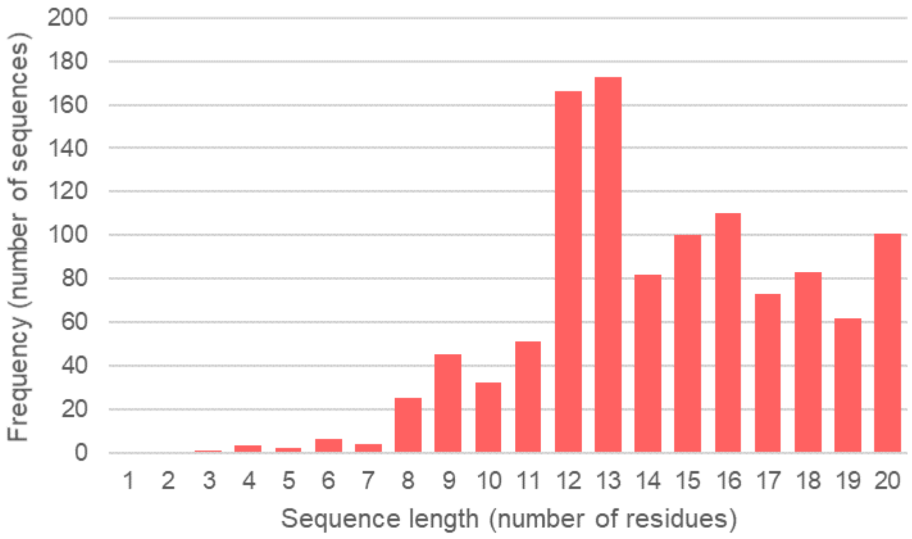

Figure S3. Histogram of the length distribution of the negative UniProt data set (n = 1119).

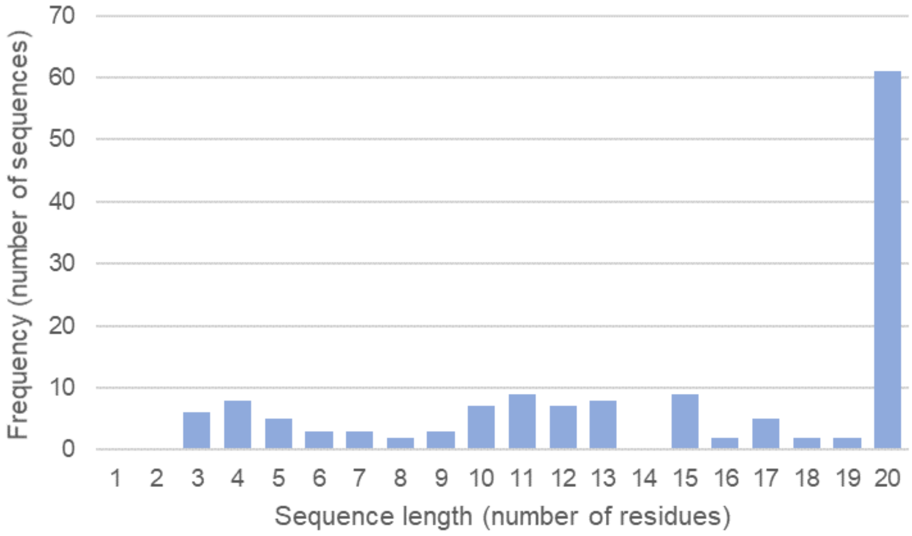

Figure S4. Histogram of the length distribution of the negative AMP (antimicrobial peptide) data set (n = 142).

Tables

Table S1. Generative model hyperparameters optimised with Bayesian hyperparameter optimization.

Generative model	Learning rate	Hidden units	Dropout rate	Loss
Sequence length ≤15 residues	0.01	512	0.0	0.3627
Sequence length ≤20 residues	0.001	480	0.0	0.2949

Table S2. Classification model hyperparameters optimised with Bayesian hyperparameter optimization.

Classification models (negative data set, MIC cut-off)	Learning rate	Hidden units	Dropout rate	Validation accuracy
Model Version 1 (AMP, ≤100 μM)	0.1	512	0.2	0.8888889
Model Version 2 (AMP, ≤50 μM)	0.1	512	0.2	0.88559324
Model Version 3 (AMP, ≤10 μM) 3	0.1	512	0.1	0.88023955
Model Version 4 (UniProt, ≤100 μM)	0.1	512	0.6	0.8526786
Model Version 5 (UniProt, ≤50 μM)	0.1	512	0.0	0.82211536
Model Version 6 (UniProt, ≤10 μM)	0.1	192	0.4	0.8158845

Table S3. Performance comparison of our classification models (marked in bold) with other state-of-the-art machine learning models. The SENS (sensitivity), SPEC (specificity), ACC (accuracy) and auROC (area under the ROC curve) are displayed in percentages.

Method	SENS (%)	SPEC (%)	ACC (%)	auROC (%)	Reference
AntiBP2	87.91	90.80	89.37	89.36	[1, 2]
CAMP-ANN	82.98	85.09	84.04	84.06	[2, 3]
CAMP-DA	87.08	80.76	83.92	89.97	[2, 3]
CAMP-RF	92.70	82.44	87.57	93.63	[2, 3]
CAMP-SVM	88.90	79.92	84.41	90.63	[2, 3]
iAMP-2L	83.99	85.86	84.90	84.90	[2, 4]
iAMPpred	89.33	87.22	88.27	94.44	[2, 5]
DNN	89.89	92.13	91.01	96.48	[2]
Multi-scale DNN	91.01	93.64	92.41	97.23	[6]
CNN	96.2	97.8	97.0	-	[7]
**Model Version 1 (AMP, ≤100 μM)**	89.3	71.4	87.3	82.3	-
**Model Version 2 (AMP, ≤50 μM)**	80.8	85.7	81.4	86.2	-
**Model Version 3 (AMP, ≤10 μM)**	89.1	86.2	88.6	90.2	-
**Model Version 4 (UniProt, ≤100 μM)**	93.8	96.9	95.3	98.2	-
**Model Version 5 (UniProt, ≤50 μM)**	94.2	93.8	94.0	98.1	-
**Model Version 6 (UniProt, ≤10 μM)**	97.1	94.2	95.7	98.1	-

Table S4. Predictions of the 14 AMP (antimicrobial peptide) sequences in the case study, by external AMP classification tools.

Peptide	AMP Scanner [2]	Deep-AmPEP30 [8]	RF-AmPEP30 [8]	iAMPpred [5]	CAMP-SVM [3]	ADAM-SVM [9]
RIHVIRWR	Y	Y	Y	Y	Y	Y
IWRVWRRW	Y	Y	Y	Y	Y	Y
APKNQLKW	N	Y	N	N	Y	Y
HRWWRWWR	Y	Y	Y	Y	Y	Y
IRRWRRIW	Y	Y	Y	Y	Y	Y
PYKISIHL	N	Y	Y	Y	N	Y
KRWWIRWR	Y	Y	Y	Y	Y	Y
APRRNVRW	Y	Y	Y	Y	Y	Y
PFKISIHH	Y	Y	Y	Y	Y	Y
RRKRWWRR	Y	Y	Y	Y	Y	Y
APLKQLKW	Y	Y	Y	N	Y	Y
PFKKSIHL	Y	Y	Y	Y	Y	Y
APWKQLKW	Y	Y	Y	Y	Y	Y
RRRRFRRR	Y	Y	Y	Y	Y	Y
**Antimicrobial (%)**	85.7	100	92.9	85.7	92.9	100

Y – predicted as active; N – predicted as inactive

References

1. Lata S, Mishra NK, Raghava GP. AntiBP2: improved version of antibacterial peptide prediction. BMC Bioinformatics. 2010;11(Suppl 1):S19. doi: 10.1186/1471-2105-11-s1-s19.
2. Veltri D, Kamath U, Shehu A. Deep learning improves antimicrobial peptide recognition. Bioinformatics. 2018;34(16):2740-7. doi: 10.1093/bioinformatics/bty179.
3. Waghu FH, Barai RS, Gurung P, Idicula-Thomas S. CAMPR3: a database on sequences, structures and signatures of antimicrobial peptides. Nucleic Acids Res. 2016;44(D1):D1094-7. doi: 10.1093/nar/gkv1051.
4. Xiao X, Wang P, Lin WZ, Jia JH, Chou KC. iAMP-2L: a two-level multi-label classifier for identifying antimicrobial peptides and their functional types. Anal Biochem. 2013;436(2):168-77. doi: 10.1016/j.ab.2013.01.019.
5. Meher PK, Sahu TK, Saini V, Rao AR. Predicting antimicrobial peptides with improved accuracy by incorporating the compositional, physico-chemical and structural features into Chou's general PseAAC. Sci Rep. 2017;7:42362. doi: 10.1038/srep42362.
6. Su X, Xu J, Yin Y, Quan X, Zhang H. Antimicrobial peptide identification using multi-scale convolutional network. BMC bioinformatics. 2019;20(1):730-. doi: 10.1186/s12859-019-3327-y.
7. Witten J, Witten Z. Deep learning regression model for antimicrobial peptide design. bioRxiv. 2019:692681. doi: 10.1101/692681.
8. Yan J, Bhadra P, Li A, Sethiya P, Qin L, Tai HK, et al. Deep-AmPEP30: Improve Short Antimicrobial Peptides Prediction with Deep Learning. Mol Ther Nucleic Acids. 2020;20:882-894. doi: 10.1016/j.omtn.2020.05.006.
9. Lee H-T, Lee C-C, Yang J-R, Lai JZC, Chang KY. A Large-Scale Structural Classification of Antimicrobial Peptides. Biomed Res Int.2015:475062. doi: 10.1155/2015/475062.
